# The card game of publish or perish: a satirical approach to the reality of academic publishing

**DOI:** 10.3325/cmj.2024.65.396

**Published:** 2024-08

**Authors:** Marko Lucijanić

**Affiliations:** 1 Hematology Department, Dubrava University Hospital, Zagreb, Croatia; 2 Scientific Research and Translational Medicine Department, Dubrava University Hospital, Zagreb, Croatia; 3 School of Medicine, University of Zagreb, Zagreb, Croatia; *markolucijanic@kbd.hr*

The phrase “publish or perish” is an aphorism that encapsulates the peer-pressure placed on researchers to constantly publish, recognized as early as 1932 (1). Scientific production is necessary to disseminate scientific ideas and improve the global pool of knowledge. It is encouraged by academic institutions, which use metrics such as the number of publications to measure the competency of individuals and credit them for their work. The pressure generated by the expectations of colleagues, institutions, and the broader academic community can drive individuals to prioritize quantity over quality and engage in undesirable behaviors (2). Just as every other form of peer pressure, publish or perish culture has consequences for the mental health of professional researchers, who, due to exposure to chronic stress, may develop burnout and anxiety, and may adopt unethical or even hazardous practices (3). It could also have consequences for the academic community, which faces erosion of research quality, unethical practices (data manipulation, plagiarism, salami publishing, etc), and a shift in priorities from teaching, mentoring, and patient-oriented practice toward the academic success of individuals and institutions. One can become a successful clinician with minimal or no publishing, but even in clinical medicine, promotions and salaries are dependent on whether one publishes or not. Thus, publish or perish practice is here to stay, and we need to develop personal coping mechanisms to deal with it.

The reality of academic publishing was somewhat humorously approached by Max Hui Bai, a social psychologist from Minnesota, USA. He created “The Publish or Perish” card game (4), which makes players compete to publish manuscripts by playing an appropriate combination of research and action cards. Every manuscript has a citation value, and the player who ultimately gains the most citations wins. The game abounds with satirical elements inspired by senseless nature and the absurdity of academic practices. These include fake titles and abstracts of made-up manuscripts, obligatory congratulation with applause to your opponents for every publication (under the threat of penalty), and a possibility to engage in dubious practices (*P* value hacking, plagiarizing data, or sabotaging opponents by audits, budget cuts, and unconstructive comments). The game also includes an expansion card pack named “Revenge of the Reviewer 2,” a title that everyone who has ever tried to publish a paper can relate to. The game is to be released in the following months (4), and has already attracted considerable attention as a “medium to socialize using the collective trauma” (5).

Games such as this can be a great learning opportunity for all of us, to relax and take the publish and perish process less seriously (the message I especially address to the Reviewer 2 of my last manuscript).
